# Durability of Concrete with Superabsorbent Polymer (SAP) Assessed Using Depth of Carbonation and NDT Ultrasonic Methods

**DOI:** 10.3390/ma17040906

**Published:** 2024-02-15

**Authors:** Joanna Julia Sokołowska

**Affiliations:** Department of Building Materials Engineering, Faculty of Civil Engineering, Warsaw University of Technology, 16 Armii Ludowej, 00-637 Warsaw, Poland; joanna.sokolowska@pw.edu.pl

**Keywords:** NDT, non-destructive quality control, direct ultrasonic method, superabsorbent polymer (SAP), concrete carbonation, durability, internal curing

## Abstract

The paper concerns destructive and non-destructive (NDT) evaluation of the effect of the addition of superabsorbent polymer (SAP) used as a carrier of mixing water and a means of internal curing on the durability of concrete. The research concerns testing of five concretes—an ordinary reference concrete and four concretes differing in the content of mixing water introduced into the concrete mix in the form of pre-saturated SAP particles (25%, two variants of 50% and 75% of the total mixing water in the form of SAP hydrogel). The research consisted of 4 stages of tests. The subsequent stages involved the analysis of the effect of using SAP as a carrier of mixing water on the particular characteristics of concrete mix and hardened concrete, i.e., consistency and density of concrete mix (1st stage), carbonation tested using two indicators—phenolphthalein and thymol phenolphthalein (2nd stage), and finally: the homogeneity of the concretes’ structure by means of ultrasonic method (determination of ultrasonic pulse velocity) 28 days after production (3rd stage) and 3 years after production (4th stage). The ultrasonic pulse (or wave) velocity was then correlated with the content of water applied in the form of SAP hydrogel. The statistical analysis of results showed that the method of introducing the mixing water into the concrete mix in the form of pre-absorbed superabsorbent polymer, although it changed the concrete mix consistency, did not significantly affect the concrete ability to resist carbonation. Meanwhile, after 3 years, the densification of the microstructure of concrete with SAP has been observed.

## 1. Introduction

Nowadays, concrete is rarely considered a three-component material; usually, apart from cement binder, water, and aggregate concrete composition includes admixtures, additives, and dispersed reinforcement (e.g., micro-reinforcement or macro fibers including, e.g., polypropylene, glass or basalt fibers [1,2]). As a result of technological development, the chemical admixtures applied to the concrete mix, even in very small amounts, can significantly improve the key properties of concrete in its fresh and/or hardened form. Despite the great achievements in the field of cement concrete modification, new solutions are constantly being invented. A relatively new idea is to modify the properties of concrete using superabsorbent polymers (SAP), which, thanks to the ability to absorb large amounts of water (even several hundred times more than their own mass), are commonly used in the production of hygiene products or water-retaining devices in agriculture and horticulture. Meanwhile, scientific papers presenting research on using superabsorbent polymers as a concrete component indicate the positive effects of SAP on concrete properties [3,4,5,6,7]. An important advantage of using SAP in concrete is the reduction of autogenous shrinkage obtained as a consequence of internal curing by means of fine-grained SAP fragments saturated with water, which gradually release water to ensure the progress of cement hydration [6,8,9,10,11]. This internal curing phenomenon was previously investigated and empirically confirmed also by the author [12].

**Figure 1:**
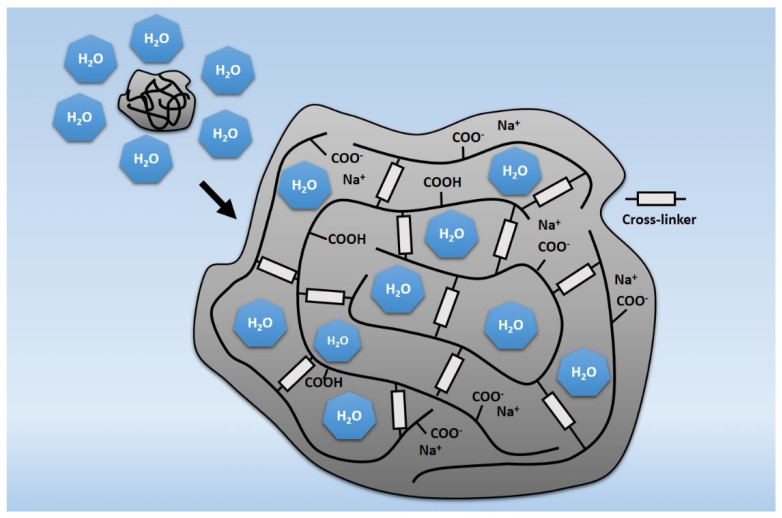
Water absorption by the SAP polymer based on polyacrylic acid (figure elaborated on the basis of [3]).

It should be noted that concrete with SAP is a relatively new solution, and so far, few results of tests carried out after a longer period have been discussed in the scientific literature—neither in the context of basic mechanical properties (destructive) tests nor in the context of non-destructive tests, which allow non-invasive assessment of whether the presence of SAP in concrete allows to create a homogeneous microstructure ensuring the durability of concrete. This paper, however, discusses the effect of introducing SAP (pre-saturated with mixing water) on its durability [13]. Typically, changes in the compressive strength of concrete, including those subjected to, for example, cyclic freezing and thawing, are selected as the durability assessment criteria. In this particular case, the measure of durability was carbonation depth and homogeneity of the concrete internal structure expressed by ultrasonic pulse velocity (in this particular case, the velocity of longitudinal wave, c_p_) over time. Superabsorbent polymers are polymers that present the ability to absorb and retain very large amounts of liquids—depending on the type, it might be up to about 1000–1500 [3,14] or even up to 5000 times more than their own mass [15], and after absorption the particles present a three-dimensional structure [16]. SAP polymers include cross-linked acrylic polyesters with acrylic acid and, more and more often, also cross-linked polyacrylate copolymers, i.e., substances that have the ability to swell in contact with water. Most recent studies focus on the development of novel superabsorbent hybrid hydrogels synthesized by e-beam radiation crosslinking [17]. Due to this property, in civil engineering, they are usually used as a layer protecting the wires or in materials intended to provide fire protection [3,6]. Superabsorbent polymers owe their ability to absorb water due to the high osmotic pressure resulting from the accumulation of trapped sodium ions in the polymer structure. The absorbed water causes the polymer to swell, simultaneously moving the Na^+^ cations away from each other (Figure 1) and reducing the pressure [3,4,16].

The SAP’s ability to absorb water is dependent not only on the osmotic pressure generated by water absorption but also on the external pressures resulting from the change in polymer volume. This property predestines SAP to be used in concrete technology—in the situations of disturbed equilibrium of the water-saturated polymer osmotic pressure and the internal stresses of the concrete, SAP particles can reduce their volume by releasing the water. This phenomenon can be treated as a method of internal curing of concrete [6,7,10,11,12] and was also in the presented research.

Modification of the properties of concrete with superabsorbent polymers is a relatively new idea. In the scientific literature, there are considerations regarding their classification as additives or admixtures. Due to the small amounts of SAP introduced into the concrete mix (less than 0.2% of the cement mass) and the method of incorporation (often together with mixing water), it seems reasonable to include them in the group of admixtures (added in an amount of ≤5% of the cement mass [18,19,20]). However, superabsorbent polymers are difficult to classify using commonly used criteria, including standard ones. Taking into account the research results published so far, SAP itself was added to concrete in very small amounts—most often not exceeding 0.3% of the cement mass [3,14]; however, analyzing the mass of SAP saturated with mixing water, i.e., values hundreds of times higher, it could be classified as a group of additives (added to the mixture in an amount exceeding the above-mentioned 5% of the cement mass).

**Figure 2:**
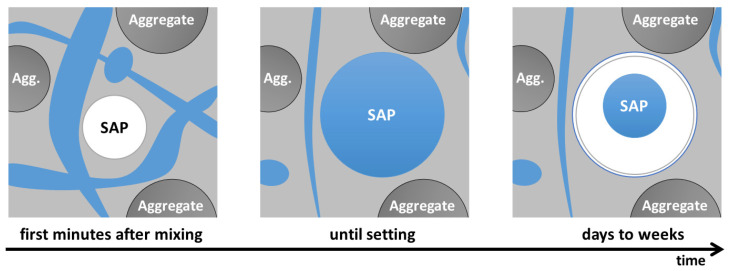
Schematic representation of desorption of SAP particle in the cement matrix over time: the SAP particle is added to the cement paste mix, and then the particle absorbs the mixing water until the matrix sets. After setting, the particle gives the water away and shrinks inside the hardened pore (figure elaborated on the basis of [4,21]).

However, the role of SAP in concrete is questionable, as it is not intended to modify the properties of the concrete mix or hardened concrete but to be a carrier of mixing water, which theoretically excludes it from the group of modifiers. However, it should be checked whether the presence of SAP affects the mechanical properties and the durability of concrete due to the absorption of part of the mixing water and the formation of a quasi-pore network filled with a polymer in the form of hydrogel [4]—Figure 2.

In the context of concrete durability assessment, researchers have attempted to reduce the damaging effects of frost by using superabsorbent polymers [5,6,21,22,23,24]. The research concept is based on the assumption that SAP can form a system of fine, evenly spaced pores, which, in concrete mix and in the “young” concrete, are filled with polymers saturated with mixing water. At a later stage of concrete maturation, as a result of using the water desorbed from the SAP particles to hydrate the cement components, empty pores are formed (Figure 2), which may act similarly to the pores resulting from the concrete mix aeration. Contrary to the unstable air bubbles obtained thanks to air-entraining admixtures, the pores derived from SAP are characterized by higher strength and thus can improve the freeze-thaw resistance of concrete [4,8,9,21].

The results of the abovementioned studies indicated that the presence of SAP in cement-based mortars subjected to cyclic freezing and thawing [5,6], including freezing and thawing in the presence of sodium chloride [22], caused no significant reduction in compressive strength. However, it might have negatively affected the flexural strength of composites (due to the size of the population of the tested specimens, it could not be clearly concluded that the strength was reduced). Moreover, composites with SAP of high water absorption capacity showed the opposite trend, i.e., the introduction of SAP particles into the concrete could be the cause of suppressing the reduction in strength.

As another positive effect of SAP application, the researchers [5,6,8,23] also indicate the limitation of the propagation of micro-cracks in the cement matrix. They explained that the higher tensile strength of the pores’ walls is a result of SAP desorption and the densification of the hydrated silicates phase (C-S-H).

## 2. Materials and Methods

### 2.1. Materials and Their Characterization

The subject of the research was concrete mixes and hardened concretes unmodified and modified with a superabsorbent polymer, which was a carrier of mixing water and a means of internal curing. All concretes were designed to obtain concrete compressive class C45/55, and the water-to-cement ratio was w/c = 0.30. The components included:

- Portland cement of high early strength, CEM I 42.5 R (meeting the requirements of EN 197-1:2011 standard [25]; for more details, see Table 1);
- Aggregate including Vistula River sand and natural gravels of fractions 2/4 mm, 4/8 mm, and 8/16 mm (meeting the requirements of the PN-EN 12620+A1:2010 standard [26] with Polish national appendix);
- Tap water (meeting the requirements of the EN 1008:2002 standard [27]);
- Superplasticizing commercial admixture (according to the manufacturer: based on modified polycarboxylates and phosphonates).

The most important properties of the applied cement binder (elaborated on the basis of the manufacturer’s data, i.e., Cementownia Ożarów cement plant (Ożarów, Poland), including selected chemical characteristics and physical properties with special attention paid to the compressive strength, are given in Table 1.

Taking into account the assumed very low value of the water-to-cement ratio (w/c = 0.30), it was necessary to use a superplasticizer, the amount of which was selected while preparing the initial concrete mixes containing large amounts of SAP. The applied superplasticizer was based on modified polycarboxylates and phosphonates. The action of such an admixture is based on the electrostatic and steric mechanisms of cement paste fluidizing. According to the manufacturer’s recommendations, the admixture dosage range was 0.3 to 3% of the cement mass. Preliminary tests enabled the determination of the optimal amount of the admixture at the level of 2.3% of cement mass.

The powdered superabsorbent polymer of declared grading 2.0/2.5 mm, marked as DMK20100 (manufacturer: DEMI Co., Ltd., Zhuhai, China) was used as a carrier of mixing water. It consisted of cross-linked polyacrylate copolymer (sodium acrylate and 2-propenoic acid polymer) (95%) and water (5%). According to the manufacturer’s recommendations, the polymer was intended for use as a water-absorbent and water-retaining agent (e.g., in sanitary devices or in agricultural and horticultural technical solutions). Information on the basic physical and chemical properties of the used polymer compiled on the basis of the technical data sheet is given in Table 2.

**Figure 3:**
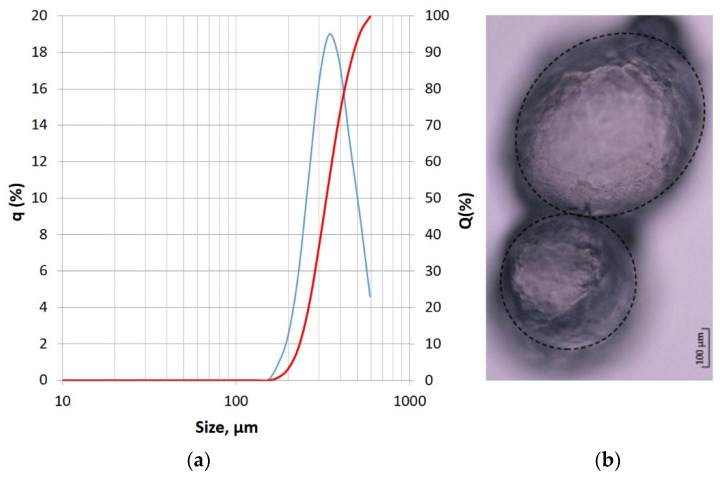
Polymer grading and morphology: (**a**) Particle size distribution plots (relative frequency plot—blue and cumulative frequency plot—red); (**b**) Morphology (micrograph taken in the digital laser microscope Keyence) [12] of the dry (unsaturated) SAP used in the tested concretes.

The manufacturer has made a very simplified particle size distribution, basically distinguishing three fractions of the dry powdered polymer. The more precise granulometric characterization was carried out using a laser diffraction particle size distribution analyzer, Horiba LA-300 (Kyoto, Japan). The measurement principles were based on laser diffraction and Mie light scattering theory (LST) [28,29]. The applied method involved passing a laser beam through a methyl alcohol with ultrasonically dispersed particles of tested superabsorbent polymer powder. The particle size distribution (PSD) was determined with a range of 0.01–600 μm. Therefore, the largest particles exceeding the 600 μm size were not recorded and are not visible in the graph presented in Figure 3.

According to the manufacturer’s technical data sheet, however, the maximum size was 2.5 mm. The real minimal recorded size was 175 µm. The distribution was unimodal, and the mode value was determined as 323 µm. Additional test output data was the specific surface area calculated on the basis of the particle size and assumption of their spherical shape. It was determined as 189 cm^2^/cm^3^. Despite some concerns regarding the accuracy of such calculations [23], in the case of tested SAP powder consisting of particles with a shape very close to spheres (confirmed by microscopic observations—see Figure 3b), it can be concluded that the values of the specific surface area obtained in abovementioned laser analyzer are close to the actual state. The detailed characteristics of the used superabsorbent polymer see [11] containing, among others, the results of the test for the absorption capacity of the SAP (set at 160 g/g in a tap water environment) and the change in dynamic viscosity of the tap water and superabsorbent polymer mix depending on the SAP/water mass ratio.

### 2.2. Concretes with SAP Subjected to Carbonation Testing and NDT

Presented research covered tests of five different concretes, including reference ordinary concrete where all mixing water was introduced in the traditional liquid form, and four concretes containing the modifier in the form of a fine-grained superabsorbent polymer. The compositions differed in the content of the SAP used, and the amount of dry SAP polymer was selected so that after complete saturation with water, the content of absorbed water was 25%, 50%, and 75% of the volume of the total mixing water. Quantitative compositions were selected based on the analysis of the influence of superabsorbent polymers on the properties of concrete, presented i.a. in [11,12].

The quantitative compositions of tested concrete covered by this paper were identical in terms of the used cement (450 kg per 1 m^3^ of concrete), aggregates (668 kg of sand, 95 kg of 2/4 mm gravel, 477 kg of 4/8 mm gravel and 668 kg of 8/16 mm gravel per 1 m^3^ of concrete), water-cement ratio, w/c = 0.30 and the amount of superplasticizer (as previously mentioned, 2.3% of the cement mass). The concretes differed in the amount of mixing water introduced into the concrete mixture bound in the superabsorbent polymer particles, i.e., in the form of SAP hydrogel (the mass of the dry polymer itself was very small and ranged around 400 ± 200 g per 1 m^3^ of concrete):

- concrete with **100% mixing water in a traditional liquid form**, no water bond in the form of SAP hydrogel—marked as **OC** (ordinary concrete);
- concrete with **25% of water bond in SAP** (75% in liquid form)—marked as **SAP-25**;
- concrete with **50% of water bond in SAP** (50% in liquid form)—marked as **SAP-50**;
- concrete with **50% of water bond in SAP** (50% in liquid form)—marked as **SAP-50s**;
- concrete with **75% of water bond in SAP** (25% in liquid form)—marked as **SAP-75**.

The compositions marked as “SAP-50” and “SAP-50s” were identical in terms of qualitative and quantitative compositions but differed in terms of the technology of concrete mix preparation. In the first case, the superplasticizer admixture was introduced into the concrete mix together with SAP hydrogel (after the “initial mixing” of hydrogel and the admixture). In the second case, a superplasticizer was applied to the mix with the “traditional” (liquid) mixing water. This additional composite SAP-50s was to validate the approach analyzed by Woyciechowski et al. in [11,12] and to roughly establish the potential (positive or negative) synergistic effects [30,31] between the superabsorbent polymer and the admixture with a highly fluidizing character [32].

### 2.3. Methods

#### 2.3.1. Concrete Mix Testing Methods

The density of the concrete mix was determined in accordance with the method described in the EN 12350-6:2019 standard [33]. The test consisted of determining the mass of compacted fresh concrete mix, filling a steel cylinder with a known volume, and calculating the volumetric density of the mix. The concrete mix consistency tests were carried out using the slump test method in accordance with EN 12350-2:2019 standard [34]. This method assumes compacting the concrete mix in the Abrams’s cone and measuring the mix slump (in the range of 10 ÷ 210 mm) after removing the mold and then matching the result to the appropriate consistency class (S1–S5).

#### 2.3.2. Carbonation Testing Method

**Figure 4:**
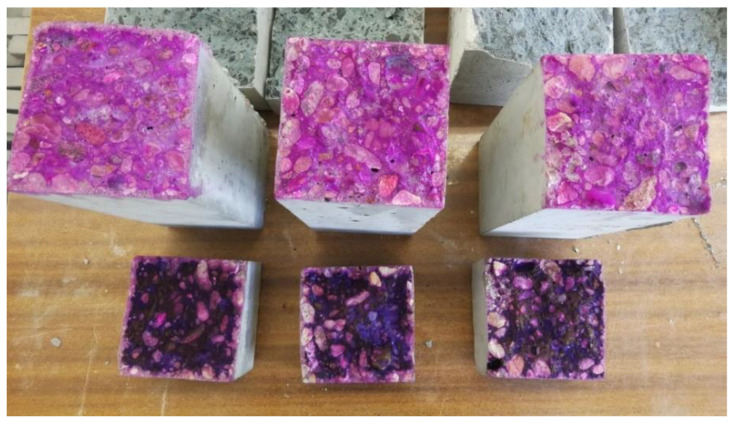
Concrete specimens are used to test the depth of carbonation with the indicator recommended in the EN 13295:2004 standard [35], i.e., phenolphthalein (indicating pH ~8.5)—upper row and thymolphenolphthalein (indicating pH ~8.5)—lower row; the uncolored area at the fracture edges indicates a carbonated area.

Concrete resistance to carbonation was tested and assessed on the basis of the procedure in EN 13295:2004 standard [35]. This method is used to test the ordinary concretes, but lately, it has also been used to assess the concretes modified with SAP [36,37]. The carbonation test each time was carried out on three cuboidal concrete specimens with dimensions of 100 mm × 100 mm × 500 mm. After 28 days of curing in water, the specimens were stored in air-dry conditions (T = 21 °C ± 2 °C, RH = 60% ± 10%) for 14 days. Then, the specimens were placed in a carbonation chamber, where they were exposed to accelerated carbonation conditions, i.e., carbon dioxide concentration of 1%, temperature of 21 °C ± 2 °C and a relative humidity of 60% ± 10%. After 14, 28, 56, and 70 days in the carbonation chamber, concrete specimens were examined in terms of the depth of carbonation. It was measured using two indicators: phenolphthalein (an indicator of pH ~8.5), i.e., the indicator that is recommended by the abovementioned standard, and thymolphenolphthalein (an indicator of pH ~9.5). At each test date, a piece of c.a. 50 mm thick concrete was broken off the specimen with a chisel, and then the abovementioned indicators were applied to the fresh fractures (Figure 4). After 60 ± 5 min, the color change to pink (or magenta) in the case of phenolphthalein and purple in the case of thymolphenolphthalein was assessed, and the depth of carbonation was measured. The uncolored area at the fracture edges indicates a carbonated area. The carbonation depth was measured at each edge of the specimen at 5 points located in its central part, 30 mm wide, ignoring the disturbances resulting from the presence of coarse aggregate. The result of the test is the average of all measurements.

#### 2.3.3. Non-Destructive Testing (NDT)

The idea of using the ultrasonic method to evaluate the durability of concrete modified with superabsorbent polymer was based on the possibility of assessing concrete homogeneity just after the recommended concrete curing time (i.e., 28 days) when the concrete should obtain the designed mechanical performance and 3 years later.

The applied method assumed the determination of the ultrasonic pulse velocity, calculated on the basis of the measurements of transit times recorded for the longitudinal ultrasonic waves passing through the concrete specimen of the known thickness (the so-called direct method). Apart from the age of composites, the tested series of concretes differed in the amount of SAP hydrogel introduced into the fresh mix (and method of superplasticizer application), which could potentially affect the homogeneity of the hardened concrete structure at the start. The ultrasonic method seemed to be a good tool to assess the quality of composites of the same age but of different SAP hydrogel content because concrete is composed of clearly separated phases of known densities, and both SAP particles saturated with water, as well as the empty spaces left after the SAP desaturation (if not covered with cement hydration products) distributed in the concrete structure, will affect the value of ultrasonic pulse velocity. It is known that the higher the volumetric density or the lower the porosity of the concrete, the higher the velocity of the wave passing through the concrete [38,39,40,41,42]. Therefore, also this aspect—the amount of water introduced to the concrete mix in the form of SAP hydrogel—was assessed in the context of the change in ultrasonic pulse velocity, thus the density of the concrete internal structure.

**Figure 5:**
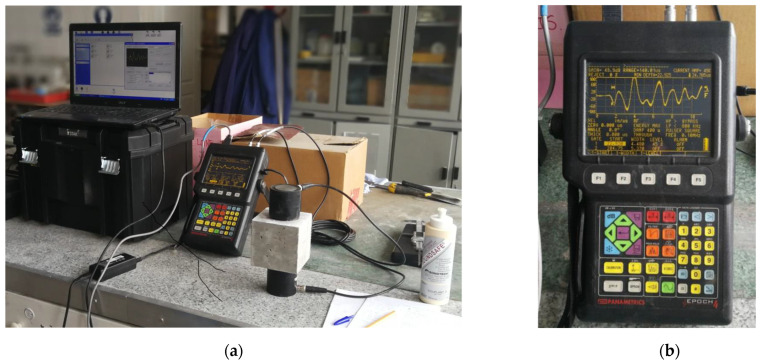
Scheme of NDT testing of concrete with SAP in digital ultrasonic flaw detector EPOCH4 Parametrics: (**a**) The measurements of transit times over a known path length (i.e., full thickness of specimen—acc. to the direct ultrasonic method) of the longitudinal ultrasonic wave; (**b**) Digital ultrasonic flaw detector screen during the measurement showing the ultrasonic wave (or ultrasonic pulse) amplitude.

The tested specimens were cubes of the size of 100 mm—each was examined by the ultrasonic method acc. to EN 12504-4:2021 standard [43] (using a variant of the abovementioned direct method [38,44]—see Figure 5) in three dimensions. Then, the average value of the ultrasonic pulse velocity (particularly longitudinal wave velocity, c_p_) was calculated. After NDT testing, specimens were left for long-term testing, and after 3 years of storage under laboratory conditions, they were again subjected to the same NDT testing. The testing was conducted using digital ultrasonic flaw detector EPOCH4 Parametrics. The measurements involved the recording of the transit times over known path lengths of the longitudinal ultrasonic wave after its transmission through the tested medium. To obtain good contact between the tested medium and heads of the signal emitter and signal receiver, layers of specialized coupling agent were applied on the concrete parallel surfaces each time.

The author wished to avoid additional variables so that comparisons of the results obtained shortly after proper curing (28 days) and after 3 years could be directly made. For this reason, the parameters of the test including, i.e., the type of emitter and receiver heads the frequency of the transducer, were accurately mapped and as follows:

- Tests were conducted using a piezoelectric transducer of a frequency of 100 kHz;
- Values of transit time were recorded for specimens of similar thickness: ca. 100 mm, being the dimension of the tested concrete cubes (measured with an accuracy of 0.01 mm);
- Transit time measurements formed the basis for calculating the ultrasonic wave velocity, which was expressed in m/s (with an accuracy of 1 m/s);

The obtained results made it possible to develop approximate trends of the ultrasonic pulse velocity changes in relation to the amount of mixing water initially introduced to the concrete mix in the form of SAP hydrogel but also in relation to the age of the tested concretes modified with SAP.

## 3. Results and Discussion

### 3.1. Concrete Mix Testing Results

The results of determining the apparent density and slump test consistency of the tested concrete mixes are presented in Table 3. The analysis of the test results did not show any significant influence of the presence of the superabsorbent polymer on the mix density. For all mixes, very similar values were obtained (population CV = 0.6%). The maximal difference between the density of concrete mix modified with SAP (SAP-25 in particular) and ordinary concrete mix was only 1.26%. These results are in line with expectations because the modification of the mix consisted only of changing the method of dosing the mixing water (from liquid water to hydrogel). Taking into account the absolutely negligible mass of the SAP polymer itself in relation to the water that it absorbs (and in relation to other components), no changes in the volume of the mix should be expected because only the geometrical arrangement of the mix components was made.

As for the concrete mix consistency, taking into account the results ranges corresponding to the consistency classes determined by the slump test method, if the permissible measurement deviations are taken into account, all tested concrete mixes can be classified into two classes: S4 or S3. Due to the absorption of part of the mixing water by the SAP particles, it was expected that the fluidity of the SAP-modified concrete mixes would deteriorate with the increase of SAP use (due to the increase in the viscosity of the blend). The obtained results are not as expected. For the SAP-25 mix and SAP-50 mix, an increase in liquidity was observed in comparison to the ordinary concrete mix—the slump test value increased by 10.8% and 21.6%, respectively. For the SAP-75 mix, a decrease in fluidity by 32.4% was observed, while for the SAP-50s mix—it decreased by 54.0% compared to an ordinary concrete result. On the basis of the obtained results, it was not possible to unequivocally determine the relation between the mix consistency and the amount of mixing water in the form of SAP hydrogel. Interesting results were obtained in the case of mixes where half of the water was pre-absorbed by polymer, differing only in the method of introducing the fluidizing admixture. The SAP-50 mix (where admixture was introduced together with water-saturated polymer) obtained the slump test value approx. 2.5 times higher than the SAP-50s mix (to which the superplasticizer was dosed with liquid mixing water directly into the mixer). Despite the significant fluidizing of the first mix, no segregation of the components was observed—the mix remained coherent.

**Figure 6:**
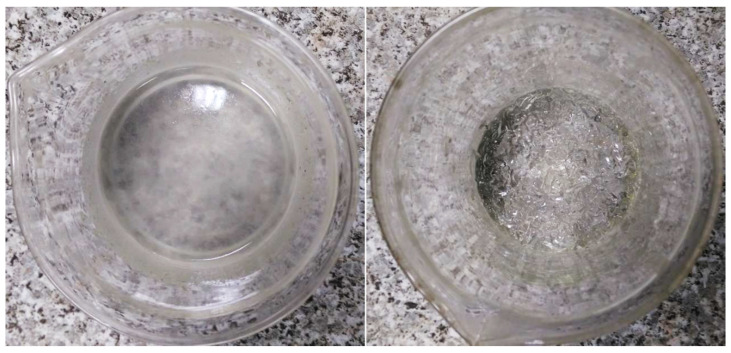
Macro-observations of SAP particles saturated with a solution of mixing water and a superplasticizer admixture (**left**) and saturated with mixing water with no admixture (**right**) show the synergistic effect between SAP and superplasticizer.

In order to confirm the synergistic effect [45,46] between the action of the superplasticizing admixture and the presence of SAP polymer, an additional test was carried out assuming the preparation of blends consisting of SAP and water or SAP, water and admixture (in the mass proportions SAP:water = 1:160). The amount of superplasticizer relative to water was the same as in the case of the previously tested concretes. The results of this additional test are presented in Figure 6.

The visual inspection of the blends clearly confirmed that there is an interaction between the superplasticizing (or fluidizing) admixture and the superabsorbent polymer. In the presence of the admixture, a clear reduction in the volume of the SAP particles was observed. The blend without the superplasticizer was clearly denser, having a ‘jelly-like’ consistency, while the blend with the superplasticizer was more fluid. Based on the above observations, it can be concluded that the simultaneous use of superplasticizers and superabsorbent polymers can reduce the absorption capacity of the second one.

### 3.2. Carbonation Testing Results

**Figure 7:**
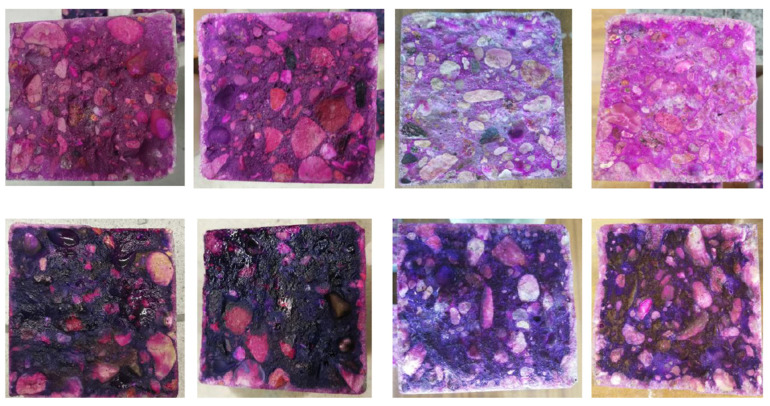
Depth of carbonation determined with the indicator recommended in the EN 13295:2004 standard [35], i.e., phenolphthalein (top row), and with an extra indicator, i.e., thymolphenolphthalein (bottom row) of concrete SAP-50 after 14, 28, 56 and 70 days (from left) in 1% of CO_2._

**Figure 8:**
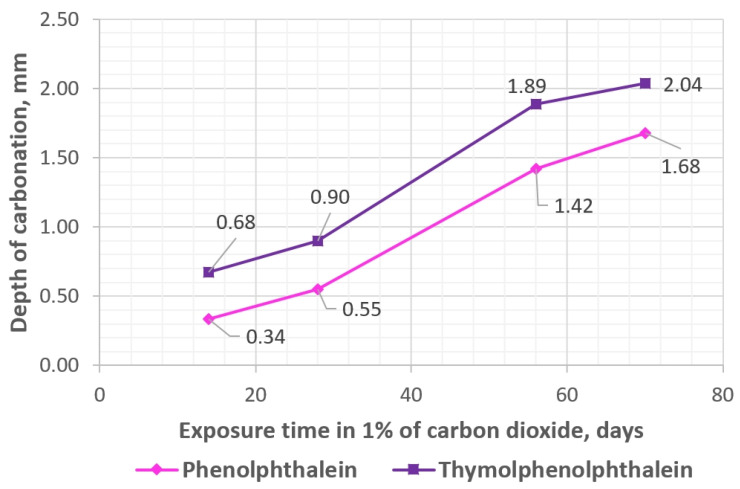
Depth of carbonation (1% of CO_2_) in ordinary concrete over time.

**Figure 9:**
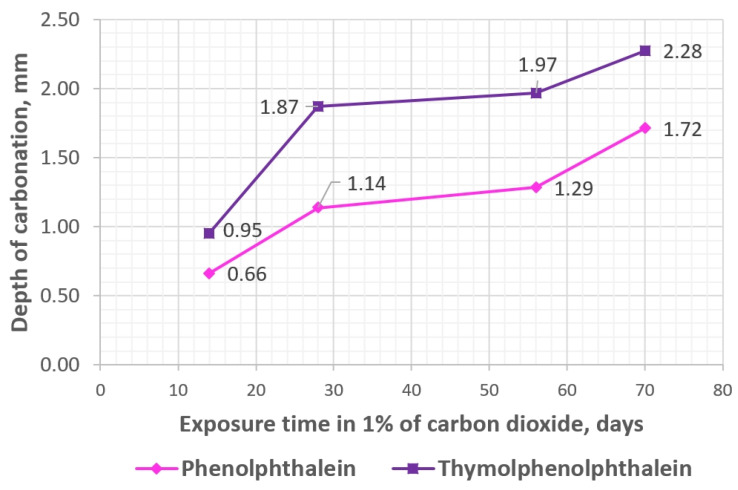
Depth of carbonation (1% of CO_2_) in concrete marked SAP-50s (50% of mixing water was introduced to the concrete mix in the form of SAP hydrogel) over time.

Carbonation depth measurements were carried out for three concretes: OC (ordinary concrete), SAP-50, and SAP-50s (concretes where 50% of mixing water was absorbed by SAP particles) at four dates—after 14, 28, 56, and 70 days in the carbonation chamber. Every time two specimens of each concrete were tested—phenolphthalein indicator was applied on one specimen, and thymolphenolphthalein indicator was applied on the other. In Figure 7, the exemplary appearance of concrete SAP-50 fractures after treatment with these indicators is shown. The non-carbonated areas of concrete marked with phenolphthalein turned pink and with thymolphenolphthalein—purple. In Figure 8 and Figure 9, one sees the development of carbonation in two abovementioned concrete—OC and SAP-50s over time (tested up to 70 days). When analyzing the course of carbonation of tested concretes, it was noticed that:

- The fastest progress of carbonation noted for OC occurred between the 28th and 56th day of the test (at other times, the depth of carbonation increased more slowly);
- In case of concretes with SAP a reverse tendency was noted: the test period between 14th and 28th day characterized by the highest carbonation development rate.

According to [36,47], the best model of the carbonation depth is the hyperbolic function of the carbonation time with the general equation:

*h*(*t*) = *a* − (*bt*)^−0.5^, (1)

where *h* is carbonation depth (in mm), *a* and *b* are material/technological coefficients (unitless), and t is exposure time, having an asymptote parallel to the time axis, which is the limit of the maximum depth of carbonation (*h_max_* = *b*). However, due to the short time of carbonation covered by the research (70 days), the attempt to find the hyperbolic pattern describing the obtained results was abandoned, and only the linear trends have been elaborated (Figure 8 and Figure 9). Nevertheless, it should be assumed that according to the state of knowledge on the phenomenon of carbonation [36,47], in the case of concrete containing SAP, this process is also terminated.

The obtained results show a slight effect of introducing the superabsorbent polymer into the concrete mix on the rate of movement of the carbonation front in the tested hardened concrete. In the case of concrete, where the admixture was introduced to concrete mix together with liquid water, practically the same results were obtained as in the case of reference ordinary concrete. In the other case (admixture introduced with pre-saturated SAP), the values of the carbonation depth were slightly higher. The greater depth of carbonation of concretes with SAP may be the result of the gradual water desorption from SAP gel particles, the formation of a pore network, and their influence on the increased diffusion of CO_2_ into the concrete structure. The obtained results are in line with the findings presented in [48,49] that the presence of SAP in concrete (with the same low value of w/c = 0.3 ratio) did not affect the course of concrete carbonation. However, in order to be able to extend the conclusions to a wider population of concretes, it is necessary to perform additional tests on composites with higher values of the water-cement ratio.

### 3.3. NDT Testing Results

The ultrasonic pulse velocity (mean values in m/s and basic statistical parameters: standard deviation, SD in m/s and coefficient of variation, CV in %) determined for ordinary concrete and for concretes with SAP are given in Table 4. The velocity (c_p_) was calculated on the basis of the measurements of transit times (in μs) recorded for concrete cubes of dimensions measured with a precision of 0.01 mm (acc. to EN 12504-4:2021 standard [43]).

Results obtained for concretes with SAP confirmed that the structure of analyzed composites had changed over time as the recorded transit times related to the distance passed by the ultrasonic wave turned out to be shorter; thus, calculated velocity changed too—after conversion, higher values of the ultrasonic wave velocity were obtained. However, the wave velocity increase was not high—up to 4.5% in the case of concretes where superplasticizer was added to concrete mix together with SAP hydrogel (see Table 4). The extreme result (an increase of 8.6%) was recorded for concrete, in which admixture was introduced to concrete mixed together with liquid mixing water. In the case of ordinary concrete, no increase in ultrasonic pulse velocity was recorded; on the contrary, the change was determined to be a decrease by 1% (probably the effect of evaporation of water that has not been bound).

**Figure 10:**
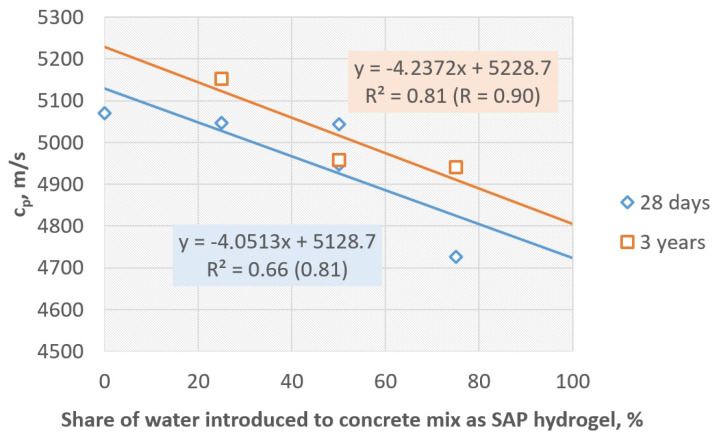
Trends describing the dependence of the ultrasonic wave velocity (c_p_) on the share of mixing water introduced to the concrete mix in the form of pre-saturated SAP hydrogel.

Taking into consideration only the concretes with SAP, where the superplasticizer was introduced in the same way (together with pre-saturated SAP), it was possible to determine trends describing the relation between ultrasonic pulse velocity and the share of mixing water introduced to the concrete mix in the form of SAP hydrogel. The trends in the form of linear functions were elaborated for the 28-day-old concretes and 3-year-old concretes (Figure 10). Both are characterized by high values of correlation coefficient (R), proving that functions were adequately adjusted to the empirical data, and high values of determination coefficient (R^2^), proving that the analyzed pulse velocity is dependent on the SAP “content”. Despite the concrete age, it was possible to indicate that the more water in the concrete mix was closed in the SAP particles at the start, the lower the ultrasonic wave velocity.

Figure 10 also shows that after 3 years the values of the ultrasonic wave velocity increased, so the additional densification of the internal structure occurred (despite the low w/c ratio of 0.30 and high mechanical strength of concretes (additional specimens were prepared from the same mixes and tested after 28 days showing compressive strength at least 66 MPa—in case of SAP-75 and maximum of 75 MPa—in case of SAP-25) resulting in even higher tightness. The average increase in pulse velocity was determined as c.a. 150 m/s—compare Table 4.

## 4. Conclusions

On the basis of the results obtained in the presented research, the following conclusions can be formulated:

- Introducing mixing water to the concrete mix in the form of pre-saturated superabsorbent polymer hydrogel (in the analyzed range, i.e., up to 75% of water) did not affect the density of the concrete mix, yet it changed the consistency. However, the unequivocal relation between the concrete consistency measured with the slump test method and the SAP hydrogel content cannot be determined.
- The influence of the superplasticizer on the SAP water absorption capacity was found. What is more, the method of introducing the superplasticizer into the concrete mix (together with SAP hydrogel or together with remaining traditional liquid mixing water) turned out to be important in the context of concrete properties.
- The obtained results showed a slight effect of introducing the superabsorbent polymer into the concrete mix on the concrete carbonation, which stands in line with the results obtained in other experiments. The greater depth of carbonation of concretes with SAP may be the result of the gradual water desorption from SAP particles, the formation of a pore network, and their influence on the increased diffusion of CO_2_ into the concrete structure.
- The applied non-destructive ultrasonic method has proved to be a sufficient and convenient tool to assess the changes in homogeneity of concrete with SAP.
- It was possible to elaborate a relation between the age of concrete modified with SAP and the densification of its microstructure resulting from the progressive internal curing. The NDT testing showed that, regardless of the direction of the ultrasonic pulse movement, very similar results of its pulse velocity were obtained within one concrete type (low CV values ranging from 0.8% to 2.3%). This proves the high degree of homogeneity of the structure of the tested concretes containing SAP.
- After 28 days of curing, the UPV values varied depending on the content of water introduced into the concrete mix in the form of SAP hydrogel, showing the general tendency that the more hydrogel, the lower the velocity. After the next 3 years, however, the values of velocity increased, confirming that the SAP did not retain water and that the later hydration performed correctly.

In response to the aim of the presented study, i.e., assessment of concrete with superabsorbent polymer durability using ultrasonic method, it can be concluded that the presence of SAP did not reduce the durability of concrete—on the contrary, the structure has become denser. Additionally, an assessment of the durability expressed by resistance to carbonation showed no significant negative effect of introducing SAP hydrogel on the carbonation depth. Therefore, the possibility of using SAP as the internal curing agent in concrete composites, previously signaled in selected literature reports, was confirmed.

## Figures and Tables

**Table 1:** Selected characteristics of the used cement CEM I 42.5 R based on the manufacturer’s data (Cementownia Ożarów, Poland).

Characteristic		Information/Value
Chemical characteristics ^1^	Ignition loss [%]	3.32
	Insoluble residue [%]	0.70
	Sulphate content SO_3_ [%]	3.13
	Chloride content Cl^−^ [%]	0.05
	Alkali content Na_2_O_eq_ [%]	0.65
Physical properties	Beginning of setting time [min]	200
	Stability of volume [mm]	0.00
	Specific surface area [cm^2^/g]	4017
Mechanical properties	Compressive strength after 2 days [MPa]	28.0
	Compressive strength after 28 days [MPa]	57.0

^1^ percentage by mass.

**Table 2:** Selected properties of the used superabsorbent polymer (SAP DMK20100) based on the manufacturer’s data (DEMI Co., Ltd., Zhuhai, China).

Characteristic		Information/Value
State of aggregation		Solid (powder)
Color		Colorless, transparent
Density, g/cm^3^		0.300–0.900
Absorbency with distilled water [g/g]		300–400
Solubility		Insoluble in water
Grading ^1^	<152 µm	≤8%
	152–750 µm	86–95%
	>750 µm	≤6%

^1^ percentage by mass.

**Table 3:** Concrete mix properties: density of compacted fresh concrete (acc. to EN 12350-6:2019 standard [33]) and consistency (tested using slump test method, acc. to EN 12350-2:2019 standard [34]).

Concrete Symbol	Properties of Concrete Mix				
	Density [kg/m^3^]	Slump Test, h [mm]	Δh ^1^ [%]	Consistency Class (Slump Test)	Consistency Class Range [mm]
OC	2452	185	-	S4	S4: (160–210) ± 30
SAP-25	2422	205	10.8	S4	
SAP-50	2457	225	21.6	S4	
SAP-50s	2431	85	−54.0	S3	S3: (100–150) ± 30
SAP-75	2425	125	−32.4	S3	

^1^ In comparison to reference ordinary concrete (OC) with no SAP.

**Table 4:** Ultrasonic wave velocity (c_p_) calculated on the basis of transit times recorded (acc. to procedure described in EN 12504-4:2021 standard [43]) 14 days and 3 years after concrete preparation.

Concrete Symbol	Ultrasonic Wave Velocity, c_p_						
	After 28 Days			After 3 Years			Change
	Mean [m/s]	SD [m/s]	CV [%]	Mean [m/s]	SD [m/s]	CV [%]	Δc_p_ [%]
OC	5070	82	1.6	5020	101	2.0	−1.0
SAP-25	5047	38	0.8	5152	211	4.1	2.1
SAP-50	4947	42	0.8	4958	107	2.2	0.2
SAP-50s	5043	55	1.1	5475	100	1.8	8.6
SAP-75	4727	111	2.3	4940	70	1.4	4.5

## Data Availability

Data are contained within the article.
